# Impact of maternal high fat diet on hypothalamic transcriptome in neonatal *Sprague Dawley* rats

**DOI:** 10.1371/journal.pone.0189492

**Published:** 2017-12-14

**Authors:** Sanna Barrand, Tamsyn M. Crowley, Ryan J. Wood-Bradley, Kirstie A. De Jong, James A. Armitage

**Affiliations:** 1 Faculty of Health, School of Medicine, Deakin University, Waurn Ponds, Victoria, Australia; 2 MMR, BCRG, School of Medicine, Deakin University, Waurn Ponds, Victoria, Australia; Universidade do Estado do Rio de Janeiro, BRAZIL

## Abstract

Maternal consumption of a high fat diet during early development has been shown to impact the formation of hypothalamic neurocircuitry, thereby contributing to imbalances in appetite and energy homeostasis and increasing the risk of obesity in subsequent generations. Early in postnatal life, the neuronal projections responsible for energy homeostasis develop in response to appetite-related peptides such as leptin. To date, no study characterises the genome-wide transcriptional changes that occur in response to exposure to high fat diet during this critical window. We explored the effects of maternal high fat diet consumption on hypothalamic gene expression in *Sprague Dawley* rat offspring at postnatal day 10. RNA-sequencing enabled discovery of differentially expressed genes between offspring of dams fed a high fat diet and offspring of control diet fed dams. Female high fat diet offspring displayed altered expression of 86 genes (adjusted P-value<0.05), including genes coding for proteins of the extra cellular matrix, particularly Collagen 1a1 (*Col1a1*), *Col1a2*, *Col3a1*, and the imprinted Insulin-like growth factor 2 (*Igf2*) gene. Male high fat diet offspring showed significant changes in collagen genes (*Col1a1* and *Col3a1*) and significant upregulation of two genes involved in regulation of dopamine availability in the brain, tyrosine hydroxylase (*Th*) and dopamine reuptake transporter *Slc6a3* (also known as *Dat1*). Transcriptional changes were accompanied by increased body weight, body fat and body length in the high fat diet offspring, as well as altered blood glucose and plasma leptin. Transcriptional changes identified in the hypothalamus of offspring of high fat diet mothers could alter neuronal projection formation during early development leading to abnormalities in the neuronal circuitry controlling appetite in later life, hence priming offspring to the development of obesity.

## Introduction

Increased intake of energy rich foods and decreased physical activity in adulthood are major factors contributing to weight gain and obesity. However, other contributing factors such as maternal high fat diet and maternal overweight or obesity during pregnancy and lactation may also predispose offspring to obesity in later life [1–5]. Animal studies suggest that maternal high fat diet during early development causes irreversible changes in the offspring hypothalamus which are associated with altered appetite control, altered sensitivity to appetite regulating hormones, and increased risk of obesity in adulthood [1, 2, 6, 7]. The hypothalamic arcuate nucleus (ARC), translates peripheral blood born signals (e.g. leptin and insulin) via orexigenic neuropeptide Y (NPY)/Agouti-related peptide (AGRP) or anorexigenic pro-opiomelanocortin (POMC)-expressing neurons. These neurons project to other hypothalamic nuclei, such as the paraventricular nuclei (PVN), to control energy homeostasis. Changes in these neuronal circuits can lead to altered feeding behaviour, as well as, altered sympathetic outflow which is a major determinant of thermogenesis [7, 8]. Modulation of these pathways is implicated in the development of obesity. For example, a decrease in the number of neuron fibres between ARC and PVN in leptin-deficient (*Lep*^*Ob*^*/Lep*^*Ob*^) mice is associated with altered food intake [9]. This phenotype can be reversed by leptin administrations between postnatal day (PN) PN4 and PN12, but not during adulthood [9]. Similarly, leptin administration between PN3 and PN13 reversed the negative impact of maternal under-nutrition on developing offspring [10]. Thus, these hypothalamic neuronal circuits may be susceptible to external environmental cues, such as maternal dietary manipulation (e.g., high fat diet, low-protein diet or under-nutrition) during early development.

Two developmental stages are thought to be particularly vulnerable to maternal nutritional manipulations (reviewed in [1, 2, 11]); (i) when the number of neurons is established between embryonic day 12 and day 14 in mice and between embryonic day 12 and day 17 in rats [1, 12, 13]; and (ii) when hypothalamic neuronal projections are formed between PN6 and PN16 in rodents [1, 14]. Maternal dietary manipulation at these time points impinges on the developing neuronal networks, further priming the offspring to increased body adiposity and metabolic disease. Indeed, offspring exposed to maternal high fat diet during pregnancy display increased density of orexigenic neurons and expression of orexigenic peptides [15]. Additionally, provision of a maternal high fat diet during lactation impairs the development of neuronal projection between hypothalamic nuclei in the offspring, which are central for energy and glucose homeostasis in mice [16]. Not surprisingly, these changes in the hypothalamus are associated with increased body adiposity, hyperleptinemia and hyperinsulinemia [16]. Maternal high fat diet and high in fat, high in sugar “junk food” diet has also been linked to altered dopamine and opioid systems that are involved in controlling food through reward and motivation [17, 18]. Thus, maternal high fat diet consumption during critical developmental windows may alter hypothalamic neuronal development; however, the molecular mechanisms by which maternal high fat diet influences hypothalamic development in offspring are currently unknown.

Changes in gene expression patterns during development may have long-term consequences for the health of an individual. Exposure to a maternal high fat diet prior to and during pregnancy is associated with alteration in the expression of neuropeptides, such as AGRP, NPY and POMC at gestational day 21 [19]. Dearden and Balthasar further demonstrated that offspring of high fat diet fed rats demonstrated a disrupted response to glucose-sensitive genes in the hypothalamus in response to insulin [20]. Other studies have linked maternal high fat diet or obesity to altered expression of neuropeptides (e.g., NPY, AGRP, and POMC) [21–25] or altered responses to leptin and ghrelin systems in the offspring [7]. Expression of dopamine-related genes in the offspring is also influenced by maternal high fat diet [18]. We aimed to determine how maternal high fat diet changes hypothalamic transcriptome at PN10; the time point when neuronal projections are forming and maturing.

## Materials and methods

### Animals and diets

All protocols in this study were approved by the Deakin University Animal Ethics Committee Geelong, Victoria, Australia. Female and Male *Sprague Dawley* rats between ages 4–6 weeks were purchased from the Animal Resources Centre, Canning Vale, WA, Australia, and allowed to acclimatise for a minimum of a week before commencement of the study. Animals were housed in pairs with an alternating light and dark cycle (12:12 hour light-dark cycle). Animals were fed either a high fat diet (HFD, 43% of calculated digestible energy from lipids, SF04-001, Specialty Feeds, Glen Forrest, WA, Australia) or control diet (CON, 12% of calculated digestible energy from lipids, SF13-081 low fat rodent diet, Specialty Feeds, Glen Forrest, WA, Australia) (S1 Table). Male rats received standard laboratory chow (Barastoc, rat and mice, Ridley, Melbourne, VIC, Australia). Average food and water consumption per day was calculated for period of 3 week (start of the diet until first day of mating), pregnancy (2 weeks following mating) and lactation (PN1 to PN10) by dividing total consumption by number of days and number of animals in a cage (ethics regulations do not allow animals to be housed individually for long period of time). To allow mating, a male rat was housed together with two female rats for a week. After mating, females were separated and housed in individual cages with environmental enrichment to facilitate natural progression of nesting and littering. Litter sizes between 6 and 16 pups were accepted for the study and litters with more than 12 pups were normalised to 12 pups at PN1, with 1:1 ratio of females and males wherever possible. This resulted in n = 6 litters for the CON dams and n = 11 litters for the HFD dams. Dams and their offspring stayed on the selected diets throughout the study and no cross-fostering was utilized. At postnatal day PN10 dams and offspring were killed humanely. Dams were killed with slow fill of CO_2_ and immediately after death blood was collected by cardiac puncture and body composition assessed using EchoMRI. Echo-MRI^TM^-100 (EchoMRI Corporation Pte Ltd, Singapore) was used to determine body composition of both dams and offspring at PN10. Rats were placed in the device individually to obtain lean mass, fat mass, free water and total water mass (g). Additionally, milk was obtained by manual expression. Mammary glands were massaged and milk was collected with a pipette after which it was transferred in a 1.5 ml tube on ice. Milk was then snap frozen and stored in -80°C until further analyses. Offspring were killed by cervical dislocation. After death, the hypothalamus was dissected from the ventral side using the anatomical landmarks of the mammillary bodies posteriorly and optic chiasm anteriorly. Hypothalami were frozen in liquid nitrogen and stored at -80°C until further analyses. Food and water were weighed three times a week throughout the study to calculate average intake.

### Body weight and body length

Throughout the study dams were weighed three times per week and percent weight change relative to start weight was determined for period of 3 week (start of diet until mating), pregnancy (start of mating until last weighing before giving birth) and lactation (PN1 to PN10) time points. Pups were weighed at PN1, PN5 and PN10. Offspring body length was measured after cervical dislocation. Body length was measured from the tip of the nose to the base of the tail, with the offspring in the supine position.

### Blood glucose and plasma leptin concentration

Blood glucose concentration for dams was determined at start of the study and after 3 weeks on the diet by obtaining small amount of blood from the tail and determining blood glucose concentration immediately using a hand-held glucometer (Accu Chek performa, Roche North Ryde, NSW, Australia). At PN10 blood was collected from dams and offspring by cardiac puncture and blood glucose concentration was determined. To obtain plasma from the offspring, the remaining exsanguinated blood was placed in Ethylenediaminetetraacetic acid (EDTA) containing tubes and centrifuged for 30 min at 1500 g, 4°C. To obtain maternal plasma, blood was placed in an EDTA containing BD vacutainer tube (cat.no 367838, Becton, Dickinson and Company, New Jersey, USA) and centrifuged for 15 min, 2500 g, 4°C. The separated plasma was removed and snap frozen in liquid nitrogen and stored at -80 ^o^C for further analysis. Plasma and milk leptin concentration were determined by ELISA according to the manufacturer’s protocol, (cat.no. 90040, Chrystal Chem, USA). Each sample was run in duplicate. Leptin concentration for all plasma samples were analysed on a single ELISA assay and all milk samples were analysed on a separate single ELISA assay. Intra-assay variability (CV%) for plasma leptin samples was 7.2% and for milk leptin samples 3.8%.

### RNA extraction

Using the manufacturer’s protocol for RNA extraction, hypothalamic RNA was extracted using a Allprep RNA/DNA mini kit (cat.no 80204 Qiagen, Hilden, Germany,) and RNA concentration and RIN number (>8 accepted) determined using a Bioanalyser (Agilent Technologies, Santa Clara, CA, USA). RNA was then aliquoted to RNAstable tubes (cat.no 93221–001 Biomatrica, San Diego, CA, USA), mixed well and dried using a speed vac. The same RNA samples were used for the RNA-sequencing and qPCR except for one sample (for qPCR) which contained insufficient RNA.

### RNA library preparation, sequencing and data analyses

A total of 24 samples (6 males, 6 females for each dietary group) were placed in RNA stable tubes and sent to an external sequencing facility (Macrogen Inc., Korea). Library preparation (TruSeq RNA Library Prep Kit) and sequencing (100bp, Illumina HiSeq 2500) was performed by Macrogen Inc., Korea. The resulting sequence results were quality checked using FastQC and trimmed (Illumina adapters) using Trimomatic. All sequences were mapped using Bowtie2 and were analysed using EdgeR, resulting in a list of significantly (FDR p = 0.05 cut-off) differentially expressed genes between the two conditions. The resulting list was further uploaded into Ingenuity (IPA) for pathway analysis. The RNA-sequencing data discussed in this publication have been deposited in NCBI's Gene Expression Omnibus and are accessible through GEO Series accession number GSE107324 (https://www.ncbi.nlm.nih.gov/geo/query/acc.cgi?acc=GSE107324)."

### CDNA synthesis, quantification and qPCR

1 ug RNA was used to prepare cDNA using a Maxima First strand cDNA synthesis kit (cat.no. FMTK1642, Thermo scientific, Waltham, Massachusetts, USA). cDNA concentration in each sample was determined using Quant-iT oliGreen ssDNA assay (cat.no. 011492, Life technologies Carlsbad, California, United States). qPCR was run in triplicates in a Thermo Scientific Pico Real Real-time PCR system. PCR efficiency for each primer was determined and only primers with primer efficiencies between 90–110% were accepted. The qPCR reaction was run using Thermo Scientific Luminaris High Green Low ROX qPCR master mix and twos-step cycling protocol (2 min 95°C, 10 min 95°C, 40x (30 sec 95°C, 60s sec 60 ^o^C)) followed by a dissociation curve. Gene expression was normalised to *Gapdh* expression and determined using the relative expression 2deltaCt method. Primer sequences were as follows:

Col1a1 F: TCAAGATGGTGGCCGTTACT R: TTGGTTAGGGTCGATCCAGT
Col1a2 F: CTGGATTTGCTGGCGAGAAG, R: AATACCGGGAGCACCAAGAAG [26]
Col3a1 F: ATGTACAGTGGCCTTCCTCA, R: TGTTTTTGCAGTGGTATGTAATGTTC [26]
Igf2 F: GTCGATGTTGGTGCTTCTCA, R: AAGCAGCACTCTTCCACGATm
LepR (ObRb) F: GAGAGGCTGCTGAAATCGTC R: CTCCAGACTCCTGAGCCATC [27]
Gapdh F: GGAAAGCTGTGGCGTGAT, R: AAGGTGGAAGAATGGGAGTT [28].

### Statistical analyses

Statistical analyses for the physiological data, mean read numbers and qPCR were analysed using 1-way ANOVA for female adult rat. A mixed model analyses was used for the offspring in IBM Statistic SPSS 22 program. Data are presented as mean ± SEM and n refers to the number of litters rather than individual pups.

## Results

### High fat diet increases body weight of adult female rat

**Fig 1:**
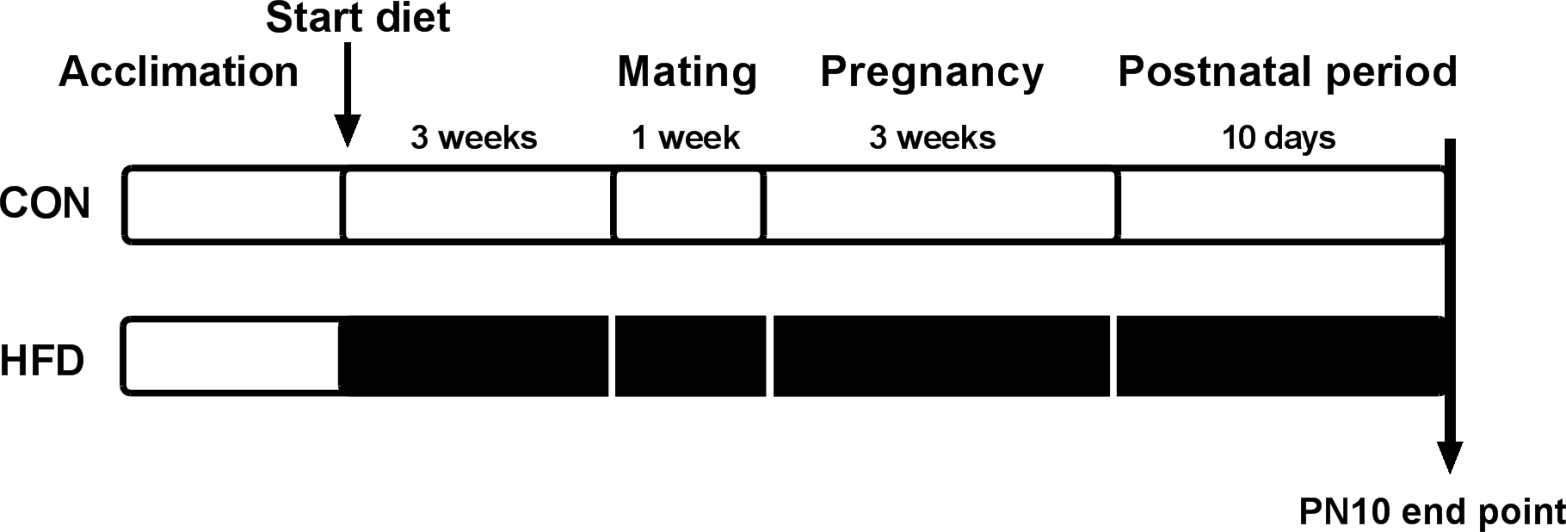
Study set up. Female *Sprague Dawley* rats received either control diet (CON) or high fat diet (HFD) throughout the study until postnatal day 10 (PN10) when offspring and dams were killed humanely and endpoint data collected.

**Fig 2:**
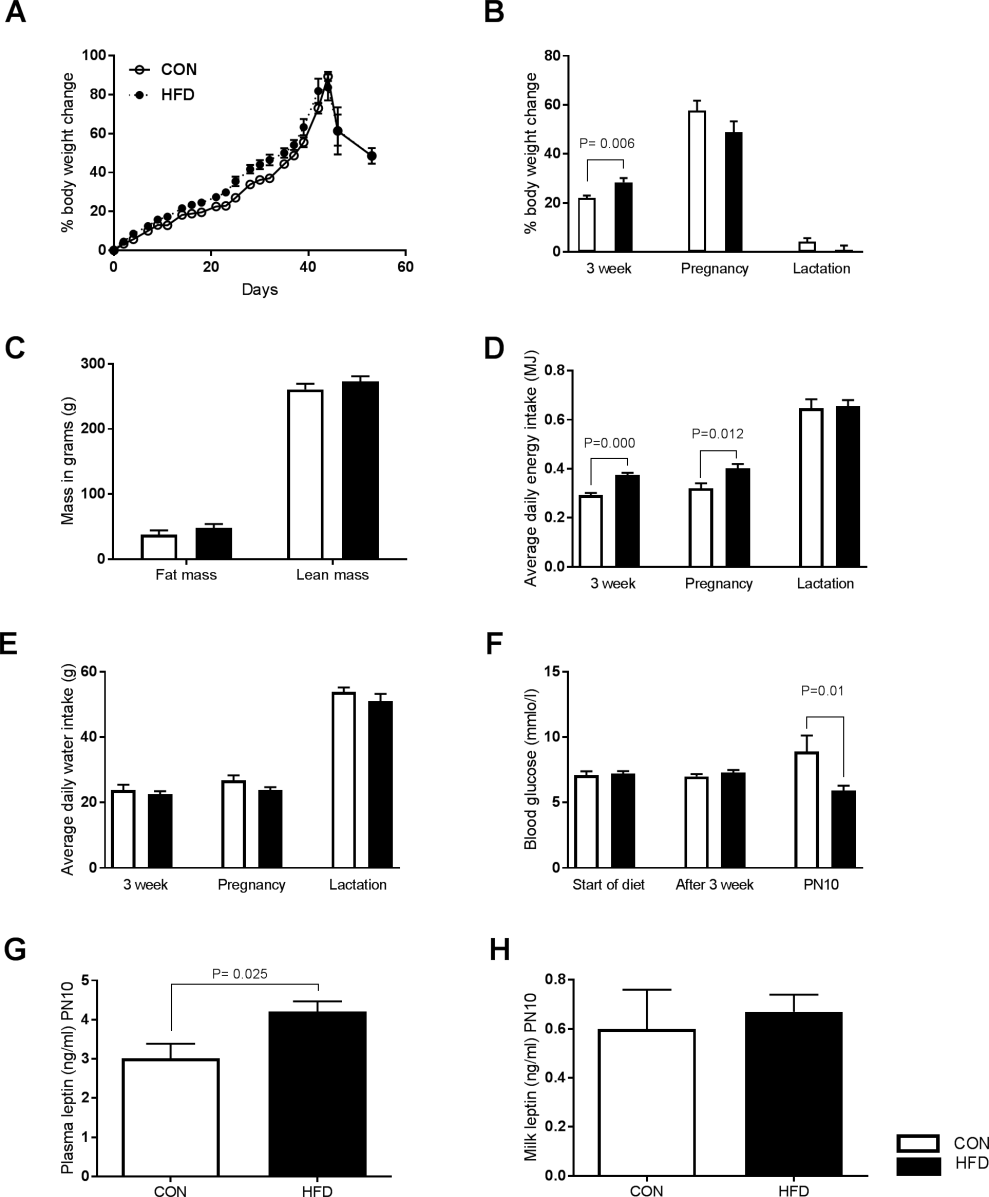
Maternal phenotype and metabolic data. (A) Illustration of maternal bodyweight changes (as percentage of weight gain to start weight) over the course of the study in HFD (filled symbol) and CON fed (unfilled symbols) females. Time points presented had CON n = 4–6 and HFD n = 8–11. (B) HFD significantly increases body weight (bodyweight change as a percentage of start weight) during the three weeks of diet, but not during pregnancy or lactation. (C) HFD does not alter fat or lean mass in HFD dams. (D) HFD dams have significantly higher average daily energy intake (MJ) during the three week diet and pregnancy but not lactation. (E) Both groups have similar water consumption. (F) Maternal blood glucose at start of diet, after 3 weeks of diet and at lactation (PN10 time point). HFD female rats have significantly lower blood glucose at lactation. (G) HFD dams have elevated plasma leptin concentrations compared with CON dams (n = 5 CON, n = 6 HFD). (H) No difference was observed in milk leptin concentrations between the group (n = 6 CON, n = 6 HFD). HFD = filled bars, CON = unfilled bars. P values are from 1-way ANOVA. Data indicate mean ± SEM. CON n = 12, HFD n = 14 for 3 week diet, CON n = 6, HFD n = 10–11 for pregnancy and lactation unless otherwise noted.

In the current study female *Sprague Dawley* rats were randomly assigned to a control diet group (CON, 12% digestible energy from lipids) or on to a high fat diet group (HFD, 43% digestible energy from lipids) 3 weeks prior to mating. The dams remained on the respective diets throughout mating, pregnancy and until the end of study at PN10 (Fig 1). HFD dams displayed significant weight gain during the 3 week diet compared to CON dams (CON 21.9±1.07 vs. HFD 28.45±0.76, P = 0.06) (Fig 2A and 2B), but no augmentation of bodyweight gain in pregnancy and during lactation (Fig 2B). In agreement with undetectable bodyweight changes during lactation we observed no significant increase in the fat mass or lean mass in HFD dams at PN10 (Fig 2C). The HFD dams had significantly greater average daily caloric intake compared to CON dams during the 3 week diet (CON 0.29±0.09 MJ/day; HFD 0.37±0.009 MJ/day P = 0.000) and during pregnancy (CON 0.32±0.02 MJ/day; HFD 0.40±0.02 MJ/day P = 0.012), but not during lactation (Fig 2D). Daily water consumption did not differ between the groups (Fig 2E). There was a significant reduction in maternal blood glucose concentration at PN10 in HFD dams compared with CON dams (CON 8.9 ±1.23 mmol/l, HFD 5.9 ±0.39 mmlo/l; P = 0.01) (Fig 2F), which was accompanied by a significant increase in maternal plasma leptin levels (CON 3.0±0.36 ng/ml, HFD 4.2±0.27ng/ml; P = 0.025) (Fig 2G). Leptin concentration in milk was similar in both groups at PN10 (Fig 2H). We detected no significant difference in the litter sizes at PN1 (before litter normalization) or at PN10 (S1 Fig) and we did not observe altered incidence of cannibalism in the HFD group. We did observe that more animals failed to become pregnant in the CON group (4 out of n = 12) than in the HFD group (n = 14). We did not assess embryonic lethality in our study.

### Maternal high fat diet feeding alters offspring phenotype and metabolic profile

**Fig 3:**
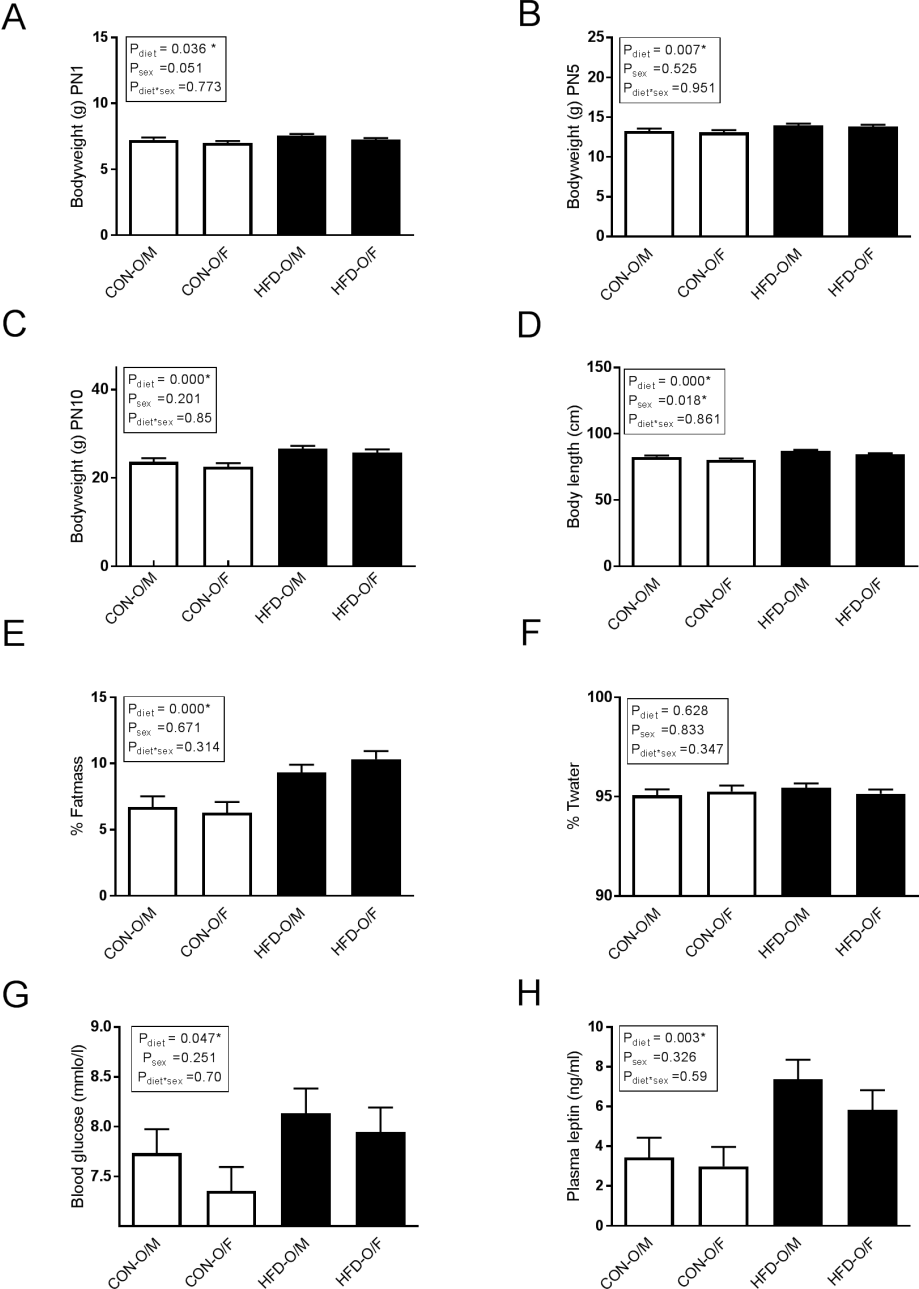
Offspring phenotype. HFD offspring are significantly heavier at (A) PN1, (B) PN5 and (C) PN10. (D) HFD offspring are significantly longer compared with CON offspring. (E) HFD offspring display increased percentage of fat mass (relative to lean mass). (F) Total water was unchanged. (G) HFD offspring have increased blood glucose at PN10 compared to CON offspring. (H) HFD offspring have increased plasma leptin at PN10 compared to CON offspring. Maternal high fat diet offspring (HFD-O) = filled bars, maternal CON offspring (CON-O) = unfilled bars. P values from 2-way mixed model ANOVA analyses. Data represent mean± SEM. CON-O (n = 6), HFD-O (n = 11) except for blood glucose and plasma leptin CON-O (n = 6) and HFD-O (n = 6). M = male, F = female.

Controlling litter size in maternal dietary models is essential since variations in litter size may directly impact nutritional availability. In our study, litter sizes between 6 and 16 pups were accepted and litters with more than 12 pups were normalized to 12 at PN1. Compared with control offspring (CON-O), the offspring of HFD dams (HFD-O) displayed significantly increased bodyweight at PN1 (P_diet_ = 0.036), PN5 (P_diet_ = 0.007) and PN10 (P_diet_ = 0.000) (Fig 3A–3C). There was no effect of sex on bodyweight. At PN10 HFD offspring were significantly longer than the CON offspring (P_diet_ = 0.000) (Fig 3D). In general, female offspring were significantly shorter than male offspring (P_sex_ = 0.018) (Fig 3D). HFD offspring displayed a significant increase in percentage of fat mass (relative to lean mass) (P_diet_ = 0.0001, P_sex_ = 0.671, P_diet_*_sex_ = 0.314) (Fig 3E), while the percentage of total water (T_water_) was unchanged between groups (Fig 3F). HFD offspring demonstrated elevated blood glucose (P_diet_ = 0.047, P_sex_ = 0.251 P_diet_*_sex_ = 0.70) (Fig 3G) and circulating plasma leptin concentrations (P_diet_ = 0.003, P_sex_ = 0.326, P_diet_*_sex_ = 0.590) (Fig 3H).

### Maternal high fat diet alters neonatal hypothalamic transcriptome at PN10

**Fig 4:**
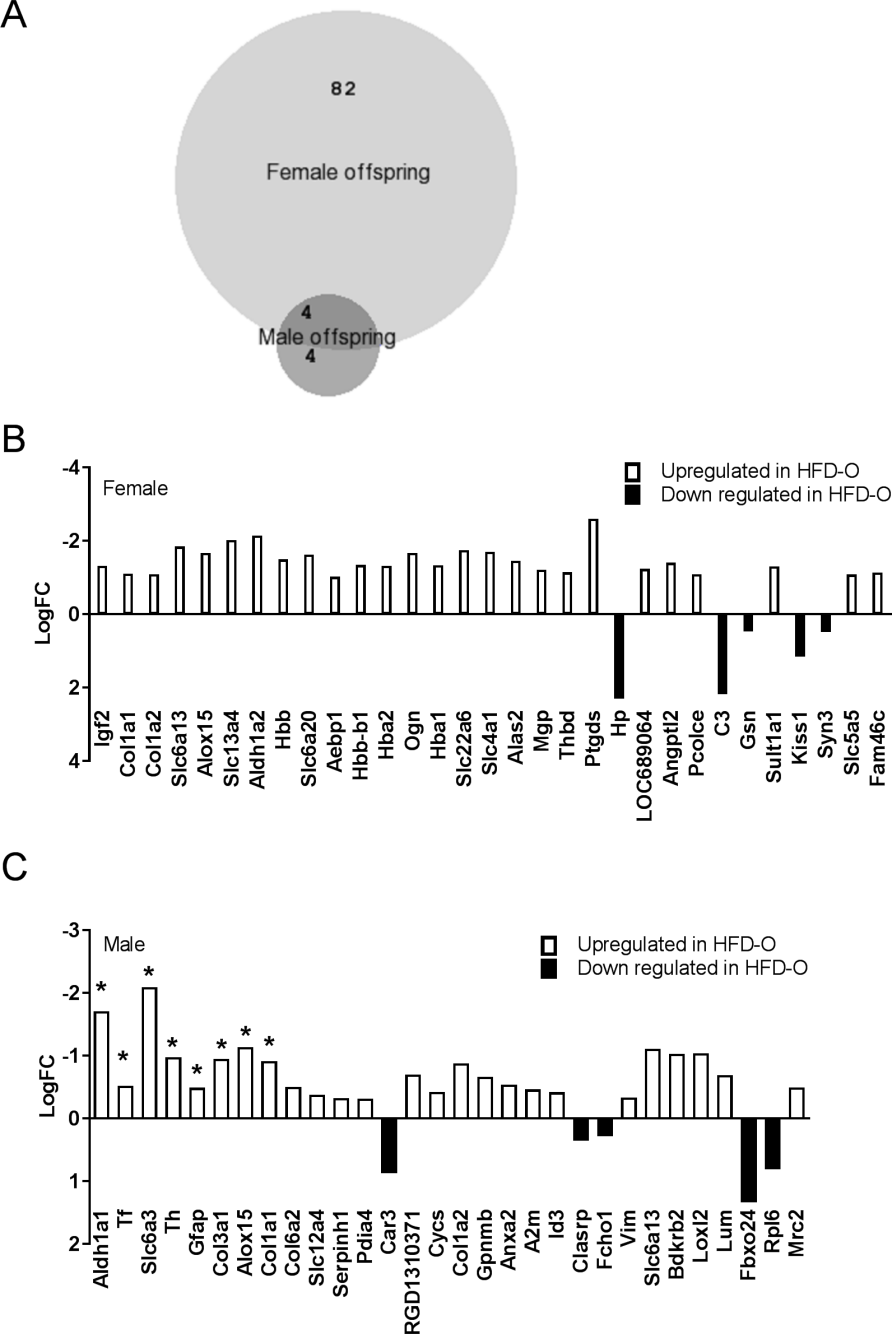
RNA-sequencing data. (A) Venn diagram illustrating differentially expressed genes in male and female offspring with stringent Adj. P-value<0.05. (B) Representation of selected differentially expressed genes in female offspring. All genes indicated have Adj.P-value <0.05. (C) Representation of selected differentially expressed genes in male offspring. All genes have P-value<0.01 or *Adj.p-value<0.05. CON-O (n = 6), HFD-O (n = 6) for both sexes. BioVenn, [31], an online web application was used to generate Venn diagrams.

Previous studies [20, 29, 30] have suggested that maternal dietary manipulation may impact the offspring in a sex specific manner and therefore we determined transcriptional changes in each sex separately. Using a stringent cut-off (adjusted P-value (adj.P-value)<0.05), we found a total of 86 differentially expressed genes in female HFD offspring compared to female CON offspring (Fig 4B). In the male offspring, we detected significant differential expression of only 8 genes in HFD offspring compared with CON offspring (adj.P-value <0.05) (Fig 4A). Of these, four genes were found differentially expressed only in male offspring and four were detected in both sexes (Fig 4A). In the female offspring, seven of the 86 differentially expressed genes were downregulated (logFC>0) and 79 were upregulated in HFD offspring compared with CON offspring (logFC<0) (S2 Table). A proportion of upregulated genes in female HFD offspring encoded for extracellular matrix (ECM) proteins, such as *Col1a1*, *Col1a2* and *Col3a1* (Fig 4B). Other differentially expressed genes included the imprinted *Igf2* gene and members of solute carriers (slc) superfamily (Fig 4B). Similarly to female offspring, male HFD offspring showed significant upregulation of *Col1a1* and *Col3a1* compared with CON offspring (Fig 4C). The four genes upregulated only in male HFD offspring were *Aldh1a1*, *Tf*, *Th* and *Slc6a3* (Fig 4C). We used Ingenuity Pathway analyses (IPA) to determine the top molecular and cellular functions associated with the differentially expressed genes in each sex (Table 1). Further we found that collagen genes and the imprinted *Igf2* gene were all associated with these cellular functions determined by IPA (Table 1).

**Table 1:** Top molecular and cellular functions identified by Ingenuity pathway analyses (IPA).

**Molecular and Cellular Functions (female ADJ.P-VALUE <0.05)**		
**Name**	**p-value range**	**Molecules of interest**
Cell Morphology	1.14E-03–6.16E-15	COL1A1, COL3A2, IGF2
Cellular Assembly and Organization	1.14E-03–6.16E-15	COL1A1, COL1A2, COL5A1, COLA3A1
Cellular Movement	1.31E-03–3.59E-12	COL1A1, COL3A1, IGF2
Cellular Function and Maintenance	1.31E-03–8.00E-12	COL1A1, COL1A2, COL3A1, COL5A1
Cell-to-Cell Signalling and Interaction	1.30E-03–4.74E-10	COL1a1, COL3A1, IGF2
**Molecular and Cellular Functions (male P-VALUE <0.01)**		
**Name**	**p-value range**	**Molecules of interest**
Cell Morphology	9.65E-04–8.68E-14	COL1A1, COL3A1
Cellular Assembly and Organization	9.63E-04–8.68E-14	COL1A1, COL1A2, COL3A1,
Cellular Function and Maintenance	8.99E-04–3.80E-12	COL1A1, COL1A2, COL3A1, IGF2
Cellular Movement	9.52E-04–9.70E-11	COL1A1, COL3A1, IGF2
Cellular Growth and Proliferation	7.73E-04–1.33E-10	COL1A1, COL1A2, COL6A2, IGF2

Genes of interest, such as *Col1a1* and *Igf2*, were identified in these pathways (male and female shown separately).

**Fig 5:**
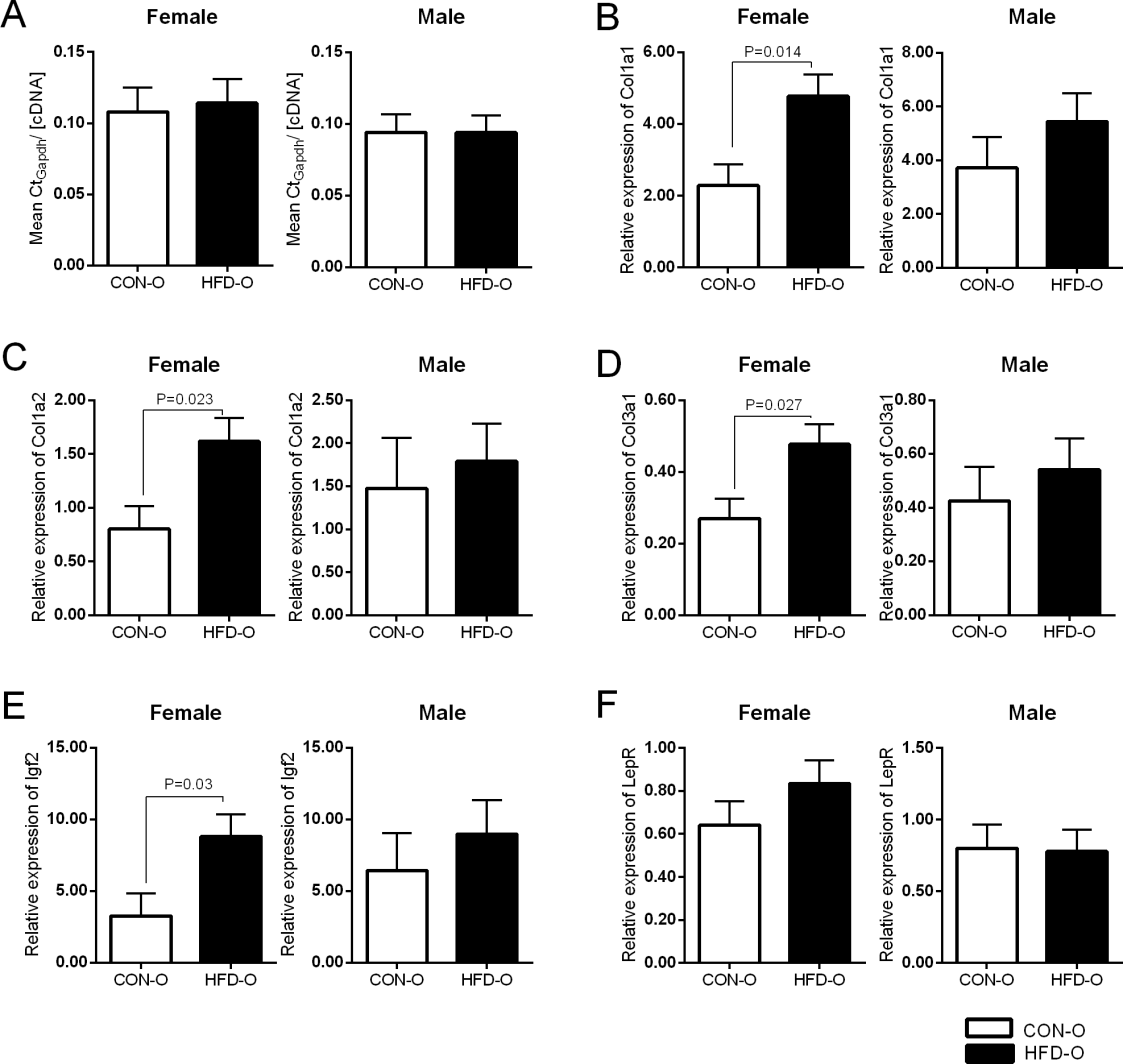
qPCR validation of selected genes from RNA-sequencing data. (A) Ratio of mean Ct_*Gapdh*_ to cDNA concertation did not changed between groups. Relative gene expression of (B) *Cola1a1*, (C) *Cola1a2*, (D) *Col3a1*, (E) *Igf2* and (F) *LepR* was determined by qPCR. All genes analysed showed similar profile to RNA-sequencing, where HFD offspring showed higher expression (significant in female offspring) in all cases except *LepR*. qPCR was run in triplicates, CON-O (n = 5–6), HFD-O (n = 6). Statistical analyses * P<0.05 in SPSS mixed model analyses. Standard errors indicated.

We validated the RNA-sequencing data for *Col1a1*, *Col1a2*, *Col3a1* and *Igf2* by real-time qPCR (Fig 5). No significant difference was detected in the ratio between mean Ct_Gapdh_ and cDNA concentration for each sample in the two dietary groups (Fig 5A) and therefore gene expression was normalised to the *Gapdh* housekeeping gene. The qPCR data confirmed upregulation of *Col1a1*, *Col1a2*, *Col3a1* and *Igf2* in female HFD offspring (Fig 5B–5E). We were unable to validate upregulation of *Col1a1* and *Col3a1* in male HFD offspring by real-time qPCR (Fig 5B and 5D). Leptin receptor (*LepR*) expression was not altered between the HFD offspring and CON offspring (Fig 5F) groups when assayed by real-time qPCR or RNA-sequencing.

## Discussion

### Maternal phenotype is altered by high fat feeding

We first determined the effect of high in fat diet consumption on adult female *Sprague Dawley* rats. We detected increased bodyweight change in the HFD dams during the 3 week diet, which can be attributed to the higher daily energy intake from to the diet. However, despite the increased energy intake during pregnancy, the HFD dams did not display a significant change in bodyweight during pregnancy. Weight changes during pregnancy could be affected by the number of pups the female is carrying, leading to larger bodyweight variations between groups. This could further mask the effect of high fat diet on bodyweight changes during pregnancy. While we did not detect significant variation in the litter sizes at PN1 or PN10, the mean litter size at PN1 showed tendency to be smaller in high fat diet group compared to control diet group (S1 Fig). While smaller litter size in HFD group could indicate increased embryonic lethality, due to our study set up, embryonic lethality was not assessed in our study. Therefore, we are unable to evaluate whether the litter sizes at PN1 were affected by embryonic lethality. Furthermore, we cannot rule out the potential effect of the litter size variation on the bodyweight during pregnancy. Further, it is important to note that bodyweight data for the 3 week included all female adult rats (even those that did not fall pregnant in the later time point), while only the animals that had successful pregnancies were included in the later time points. This could potentially contribute to the difference seen in the bodyweight results between 3 week diet and pregnancy.

At the PN10 endpoint HFD dams exhibited increased plasma leptin concentration. Leptin is expressed in the white adipose tissue and circulating leptin levels are found to increase with increasing body adiposity. We were unable to detect difference in the fat mass between the two dietary groups, despite the fact that the HFD dams exhibited increased plasma leptin. This suggests that small elevations in body fat could be the source of increased plasma leptin in these animals, or perhaps that this fat is showing greater endocrine activity per unit of mass. Maternal high fat diet consumption is also reported to alter leptin concentration in the breast milk, however the results vary greatly [16, 32, 33]. For example, Sun et al. showed that maternal high fat feeding was associated with increased maternal plasma leptin, but had no effect on milk leptin concentration at PN10 [32]. The same study further demonstrated increased concentration of leptin in the milk at PN21 [32]. Vogt et al also demonstrated increased milk leptin concentration in high fat diet consuming dams at PN19 [16]. Conversely, no impact of maternal high fat diet to milk leptin levels at PN21 was reported by Purcell et al. [33]. Similarly to study reported by Sun et al.[32], we were unable to detect changes in the milk leptin concentration at PN10, despite the elevated maternal plasma leptin levels. The extent to which the milk leptin level is altered by maternal diet, could therefore be dependent on the maternal diet and on the time point when milk is analysed during lactation.

At PN10 time point HFD dams displayed reduced blood glucose. During lactation, mothers have increased energy requirements to support infant growth. In our study HFD dams reduced their energy intake during lactation to the level of the CON dams, which indicates that their food intake (adjusted for weight) decreased during the postnatal period. HFD offspring were also found to be larger and could therefore be anticipated to have higher energy requirements and potentially consume more milk to meet these requirements when compared to CON offspring. Others have shown that high fat diet mothers spend more time nursing and grooming, while their offspring exhibit increased milk consumption [33]. We did not investigate the feeding behaviour of offspring in our studies and therefore are unable to conclude whether this would contribute to decreased maternal feeding and blood glucose after birth or whether other factors, such as the well documented postpartum hypoglycaemic episodes experienced by diabetic women played role in this.

### Offspring phenotype and metabolic parameters

Previous studies have extensively shown that maternal dietary manipulation has long lasting effects on offspring health. We observed increased body length, weight and adiposity in the offspring of maternal HFD at PN10. Additionally, HFD offspring displayed increased blood glucose and circulating plasma leptin indicating that HFD pups have impaired metabolic function. Previous studies have reported sex-specific effect of maternal diet [20, 29, 30]. We failed to observe a significant interaction between diet and sex upon plasma leptin and blood glucose concentration, suggesting that maternal HFD did not have a sex-specific effect on the offspring metabolic profile at this early time point. However, we cannot rule out the possibility that other parameters, such as plasma lipids, which were not explored in this study, could display sex-specific traits in these animals. Additional studies in maternal dietary models should be encouraged to increase our understanding of sex-specific impacts of maternal diet.

Our investigation into transcriptional changes in the neonatal hypothalamus found that maternal high fat diet altered expression of genes coding for extracellular matrix (ECM) proteins. Components of ECM in the brain include proteoglycans (e.g., agrin), glycoproteins (e.g., reeling) and fibrous glycoproteins (e.g. collagens) [34]. ECM proteins have been suggested to regulate neural development, survival, and migration and synapse formation (reviewed in [34]). Collagens are a family of extracellular and transmembrane proteins that commonly consist of three alpha-chains organised in helical structures. During development collagens may play important role in axon guidance, synaptogenesis and brain architecture [35]. We detected changes in *Col1a1* and *Col3a1* in both female and male HFD offspring. Additionally, other ECM proteins, namely laminin subunit gamma3 (*lamc3*) and fibronectin 1 (*fn1*), were significantly changed in the female offspring, further suggesting role for ECM components in hypothalamic development. There is a lack of data defining the roles for the ECM components, particularly collagens, in hypothalamic development during early postnatal life in maternal high fat diet models. Interestingly, altered expression of collagens have been identified in other tissues in a postnatal overfeeding model [36]. In this study, increased collagen expression was demonstrated in the offspring’s heart. The gene expression changes were associated with increased body adiposity, plasma cholesterol, leptin and insulin levels, as well as, altered cardiovascular function at 7 months old suggesting that dietary manipulation during early development may have long lasting effects on the health of the next generation [36].

We did not detect changes in the transcription of hypothalamic appetite regulatory neuropeptides (e.g., NPY, AgRP and POMC). Interestingly, previous reports of the gene expression levels of the appetite regulatory pathways vary greatly between studies [16, 37, 38]. For example, Morris and Chen 2008, showed that maternal high fat diet before and during gestation led to decreased expression of NPY and POMC in PN1 offspring [37], while in a postnatal over feeding model offspring exhibited increased NPY and AgRP transcripts in PN24 [38]. Thus, impact of maternal diet on hypothalamic transcriptome may be dependent on timing of intervention, dietary model used, as well as, the timing of the mRNA analyses. In our model, dams received high fat diet from 3 weeks prior mating, during pregnancy and lactation and until PN10 end point. Therefore, transcriptional changes observed in our study may have origins from gestational period, postnatal period or both. Notwithstanding, we aimed to determine the impact of maternal high fat diet on hypothalamic transcriptome at time when neuronal circuits are developing at around PN10. We believe that our study has highlighted important transcriptional changes in hypothalamus of the offspring of high fat diet consuming dams during a critical developmental time window. In addition, consideration should be given to our choice of using whole hypothalamus rather than hypothalamic nuclei in our study. Heterogeneity of tissues has previously been suggested to have potential to impact ability of RNA-sequencing to estimate abundancy of transcripts [39]. Yet this may be possible, use of whole hypothalamic extract in our study ensured material availability for RNA-sequencing. We found that the real time qPCR data correlated well with the RNA sequencing results in the female offspring. Conversely, we were unable to validate the increased expression of *Col1a1* and *Cola3a1* detected by RNA-sequencing in the male offspring. This could be due to the methodological differences between RNA sequencing and qPCR (reviewed in [40]), or due to the fact that we had one less sample in the male CON offspring in qPCR (n = 5 not n = 6). This could contribute to difference in results between RNA-sequencing and qPCR. We believe our data identifies changes in the neonatal hypothalamic transcriptome that may ultimately be the result of maternal high fat consumption.

Interestingly we observed upregulation of the imprinted *Igf2* gene in the female HFD offspring. The imprinted growth promoting *Igf2* gene is expressed highly during early development and is important for normal brain development (reviewed in [41]). In a *Zebrafish* model, disruption of *Igf2* expression is associated with abnormal brain structures [42]. Previous studies have demonstrated that maternal dietary manipulations alter transcriptional status of imprinted genes [43–45]. We observed significant increase of two imprinted genes, namely *Igf2* and Cyclin-Dependent Kinase Inhibitor 1C (*Cdkn1c*), in the female HFD offspring compared to CON offspring. Maternal low protein diet has been shown to increase expression of *Cdkn1c* mRNA in the offspring [25]. Interestingly, both *Igf2* and *Cdkn1c* have been suggested to have a role in dopaminergic neuron differentiation [46–48]. The dopamine system consists of neurons in several brain regions, including ventral tegmental area, substantia nigra and hypothalamus. In our study increased expression of *Cdkn1c* could indicated that maternal high fat diet consumption impacts hypothalamic dopamine neurocircuitry, however the behavioural outcome of this programming warrants further investigation. Vucetic *et al*. showed that maternal consumption of low protein diet during pregnancy and lactation altered *Th* and *Dat1* expression in several brain regions in offspring [25]. In agreement with this study we observed changes in expression of the two genes involved regulation of dopamine availability, namely *Th* and *Slc6a3* (also known as *Dat1*). Interestingly, these changes were only significant in the male HFD offspring. Our data suggest that maternal high fat consumption may impact hypothalamic dopamine system in hypothalamus at PN10 in a sex-specific manner. How these transcriptional changes may contribute to hypothalamic programming will need to be addressed in further studies.

In summary, our data shows that maternal high fat diet consumption alters both phenotype and metabolic status of the offspring at PN10. These changes are associated with gene expression changes in the hypothalamus. We observed increased expression of collagen genes in both sex, while other genes, such as imprinted *Igf2* gene and dopamine-related genes *Th* and *Dat* were altered in a sex-specific manner. Further studies are needed to assess the exact role of these genes in the developing hypothalamus and whether these events may contribute to development of obesity in later life.

## Supporting information

S1 Table Nutritional parameters of the diets. (PDF)

S2 Table List of differentially expressed genes in female and male offspring. (PDF)

S1 Fig Litter sizes at PN1 and PN10. (TIF)
