# Supplementary material for: Impact of maternal high fat diet on hypothalamic transcriptome in neonatal Sprague Dawley rats
Source: PLoS One. 2017 Dec 14;12(12):e0189492. doi: 10.1371/journal.pone.0189492 (PMC5730210; doi:10.1371/journal.pone.0189492)
Supplement: S1 Table — (PDF) [file pone.0189492.s001.pdf]

Calculated Nutritional Parameters<br>(% indicates values v/w)	HFD	CON
Protein	22.60%	22.60%
Total Fat	23.50%	5.30%
Crude Fibre	5.40%	5.40%
AD Fibre	5.40%	5.40%
Digestible Energy	19 MJ / Kg	15.4 MJ / Kg
Energy contribution for non-fibre carbohydrates	6.56 MJ/Kg (34.5%)	9.50 MJ/Kg (61.7%)
Total calculated digestible energy from lipids	43.00%	12.00%
Total calculated digestible energy from protein	21.00%	25.80%
**Ingredients**	**HFD**	**CON**
Casein (Acid)	233 g/Kg	233 g/Kg
Sucrose	201 g/Kg	201 g/Kg
Lard	207 g/Kg	23 g/Kg
Soya Bean Oil	29 g/Kg	29 g/Kg
Cellulose	58 g/Kg	58 g/Kg
Wheat Starch	92 g/Kg	276 g/Kg
Dextrinised Starch	117 g/Kg	117 g/Kg
L Methionine	3.5 g/Kg	3.5 g/Kg
Calcium Carbonate	6.4 g/Kg	6.4 g/Kg
Sodium Chloride	2.6 g/Kg	2.6 g/Kg
AIN93 Trace Minerals	1.6 g/Kg	1.6 g/Kg
Potassium Citrate	19.2 g/Kg	19.2 g/Kg
Dicalcium Phosphate	15.1 g/Kg	15.1 g/Kg
Potassium Sulphate	1.6 g/Kg	1.6 g/Kg
Choline Chloride (75%)	1.3 g/Kg	1.3 g/Kg
AIN93 Vitamins	12 g/Kg	12 g/Kg
**Calculated Fatty Acid Composition**<br>(% indicates values v/w)	**HFD**	**CON**
Saturated Fats C12	0 or less Trace	0 or less Trace
Myristic Acid 14:0	0.30%	0.04%
Palmitic Acid 16:0	5.80%	0.90%
Stearic Acid 18:0	3.70%	0.50%
Other Saturated Fats	0.10%	0.03%
Palmitoleic Acid 16:1	0.40%	0.05%
Oleic Acid 18:1	7.70%	1.50%
Gadoleic Acid 20:1	0.20%	0.02%
Linoleic Acid 18:2 n6	4.46%	1.80%
Linolenic Acid 18:3 n3	0.49%	0.20%
EPA 20:5 n3	No data	No data

DHA 22:6 n3	No data	No data
Total n3	0.52%	0.23%
Total n6	4.50%	1.82%
Total Mono Unsaturated Fats	8.24%	1.53%
Total Poly Unsaturated Fats	5.11%	2.07%
Total Saturated Fats	10.03%	1.50%
