# Supplementary material for: Impact of maternal high fat diet on hypothalamic transcriptome in neonatal Sprague Dawley rats
Source: PLoS One. 2017 Dec 14;12(12):e0189492. doi: 10.1371/journal.pone.0189492 (PMC5730210; doi:10.1371/journal.pone.0189492)
Supplement: S2 Table — (PDF) [file pone.0189492.s002.pdf]

Female differentially expressed genes, adj.P-value<0.05			
Name	logFC	PValue	adj.p.value
Igf2	-1.323	1.4E-15	2.0E-11
Col1a1	-1.104	2.3E-14	1.1E-10
Col1a2	-1.095	2.9E-13	8.9E-10
Slc6a13	-1.848	4.8E-12	1.1E-08
Alox15	-1.670	6.8E-12	1.4E-08
Col3a1	-1.008	4.0E-11	7.1E-08
Slc13a4	-2.023	1.2E-10	1.9E-07
Aldh1a2	-2.143	6.1E-10	7.9E-07
Hbb	-1.495	7.4E-10	8.7E-07
Ifi27	-0.890	1.0E-09	1.1E-06
Slc6a20	-1.627	1.4E-09	1.3E-06
Aebp1	-1.020	1.4E-09	1.3E-06
Hbb-b1	-1.345	1.9E-09	1.6E-06
Hba2	-1.321	3.0E-09	2.3E-06
Ogn	-1.670	5.6E-09	3.4E-06
Hba1	-1.340	5.6E-09	3.4E-06
Slc22a6	-1.749	6.1E-09	3.6E-06
Slc4a1	-1.701	1.2E-08	6.5E-06
Cdkn1c	-0.717	1.2E-08	6.5E-06
Bgn	-0.694	2.3E-08	1.2E-05
Alas2	-1.459	8.0E-08	3.5E-05
Gjb2	-0.948	1.6E-07	6.4E-05
Mgp	-1.214	1.6E-07	6.4E-05
Thbd	-1.143	1.7E-07	6.4E-05
Fbln1	-0.722	3.7E-07	1.3E-04
Colec12	-0.996	4.5E-07	1.6E-04
Ptgds	-2.597	7.1E-07	2.3E-04
Slc12a4	-0.479	7.2E-07	2.3E-04
Gjb6	-0.854	8.0E-07	2.5E-04
Hp	2.307	1.5E-06	4.5E-04
LOC689064	-1.233	1.9E-06	5.3E-04
Angptl2	-1.399	1.9E-06	5.5E-04
Cxcl12	-0.427	3.2E-06	8.9E-04
Serpinh1	-0.319	4.7E-06	1.3E-03
Col5a1	-0.645	5.7E-06	1.5E-03
Pcolce	-1.085	1.1E-05	2.7E-03
Dcn	-0.966	1.4E-05	3.2E-03
Nbl1	-0.369	1.4E-05	3.2E-03
Cfh	-0.495	1.5E-05	3.4E-03
C3	2.179	1.7E-05	3.6E-03
Ghdc	-0.456	2.3E-05	4.9E-03

Lamc3	-0.652	2.6E-05	5.4E-03
Sult1a1	-1.302	2.9E-05	5.8E-03
Gsn	0.468	2.9E-05	5.8E-03
Fn1	-0.405	3.4E-05	6.7E-03
Ctss	-0.402	3.4E-05	6.7E-03
Txnip	-0.353	3.5E-05	6.7E-03
Mrc1	-0.796	5.3E-05	1.0E-02
Col6a2	-0.457	6.4E-05	1.2E-02
Kiss1	1.162	6.7E-05	1.2E-02
Efemp1	-0.930	6.7E-05	1.2E-02
Syn3	0.484	7.1E-05	1.3E-02
Ptrf	-0.466	7.2E-05	1.3E-02
Slc5a5	-1.081	7.8E-05	1.4E-02
Gfap	-0.346	7.8E-05	1.4E-02
LOC287167	-1.014	8.0E-05	1.4E-02
Cpeb4	0.339	8.3E-05	1.4E-02
Rsad2	-0.977	9.6E-05	1.6E-02
Avp	-0.499	1.0E-04	1.6E-02
Lum	-0.689	1.1E-04	1.8E-02
Fcho1	0.275	1.1E-04	1.8E-02
Nid2	-0.458	1.2E-04	1.8E-02
Mfap4	-0.549	1.3E-04	1.9E-02
Vwf	-0.368	1.4E-04	2.2E-02
Egflam	-0.503	1.6E-04	2.4E-02
Mrc2	-0.469	1.7E-04	2.6E-02
Kank2	-0.446	1.8E-04	2.6E-02
Vtn	-0.404	1.8E-04	2.6E-02
Pltp	-0.279	1.9E-04	2.8E-02
Man2b2	-0.555	2.1E-04	3.0E-02
Fgl2	-0.791	2.2E-04	3.0E-02
Olfml2a	-0.357	2.2E-04	3.0E-02
Rarres2	-0.939	2.2E-04	3.0E-02
Fstl1	-0.326	2.3E-04	3.1E-02
Cdc25b	-0.480	2.5E-04	3.2E-02
Fam46c	-1.137	2.8E-04	3.6E-02
Anxa5	-0.318	2.9E-04	3.7E-02
Slc2a12	-0.477	3.0E-04	3.7E-02
Slc16a11	-0.611	3.0E-04	3.7E-02
Rpp40	-0.739	3.2E-04	3.8E-02
Slc9a2	-0.969	3.2E-04	3.8E-02
Myh9	-0.283	3.4E-04	4.1E-02
Dab2	-0.416	3.5E-04	4.2E-02
Fau	-0.360	3.7E-04	4.3E-02

Serpinb6b	-0.945	4.0E-04	4.6E-02
Serping1	-0.678	4.4E-04	4.9E-02

Male differentially expressed genes, adj.P-value<0.05

Name	logFC	PValue	adj.p.value
Aldh1a1	-1.709	6.8E-11	9.6E-07
Tf	-0.522	1.6E-06	5.4E-03
Slc6a3	-2.093	1.9E-06	5.4E-03
Th	-0.978	1.9E-06	5.4E-03
Gfap	-0.492	2.3E-06	5.4E-03
Col3a1	-0.951	5.0E-06	1.0E-02
Alox15	-1.140	1.9E-05	2.9E-02
Col1a1	-0.917	2.6E-05	3.6E-02
