# Supplementary figures and images for: Impact of maternal high fat diet on hypothalamic transcriptome in neonatal Sprague Dawley rats
Source: PLoS One. 2017 Dec 14;12(12):e0189492. doi: 10.1371/journal.pone.0189492 (PMC5730210; doi:10.1371/journal.pone.0189492)

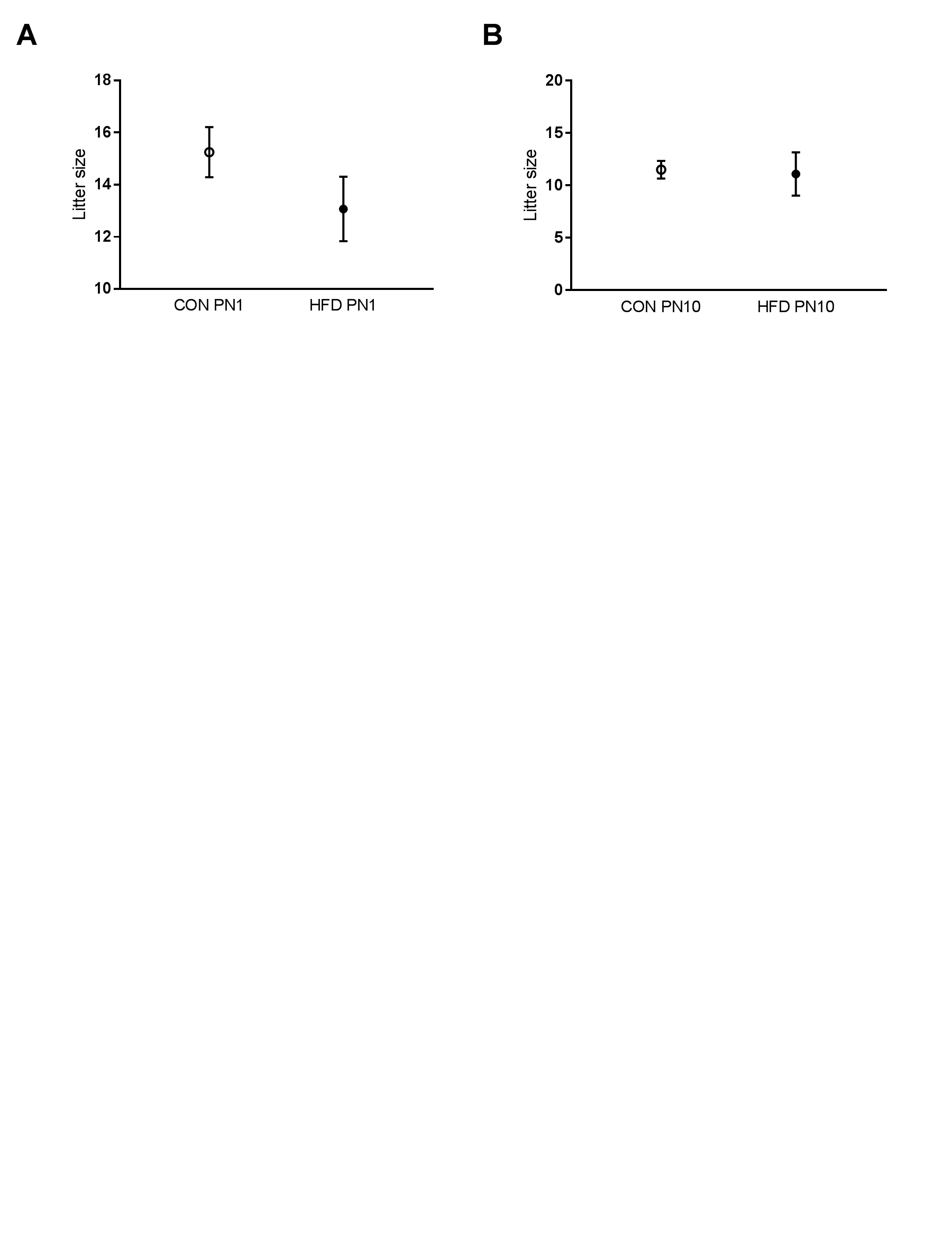

Supplement: S1 Fig — (TIF) [file pone.0189492.s003.tif]
